# Direct insertion of an ion channel immobilized on a soft agarose gel bead into a lipid bilayer: an optimized method

**DOI:** 10.1007/s44211-025-00792-y

**Published:** 2025-05-20

**Authors:** Mami Asakura, Shuyan Wang, Minako Hirano, Toru Ide

**Affiliations:** 1 Graduate School of Interdisciplinary Science and Engineering in Health Systems, Okayama University, 3-1-1 Tsushima-naka, Kita-ku, Okayama-shi, Okayama 700-8530 Japan; 2 Department of Comprehensive Technical Solutions, Okayama University, 3-1-1 Tsushima-naka, Kita-ku, Okayama-shi, Okayama 700-8530 Japan

**Keywords:** Ion channel, Artificial lipid bilayer, Suction fixation, Soft agarose bead, Current recording

## Abstract

In this paper, we report the development of a device that improves the conventional artificial lipid bilayer method and can measure channel currents more efficiently. Ion channel proteins are an attractive research target in biophysics, because their functions can be measured at the single-molecule level with high time resolution. In addition, they have attracted attention as targets for drug discovery because of their crucial roles in vivo. Although electrophysiological methods are powerful tools for studying channel proteins, they suffer from low measurement efficiency and require considerable skill. In our previous paper, we reported that by immobilizing channel proteins on agarose gel beads and forming an artificial lipid bilayer on the bead surface, we simultaneously solved two problems that had been hindering the efficiency of the artificial bilayer method: the time-consuming formation of artificial lipid bilayers and the time-consuming incorporation of channels into artificial bilayers. Previous studies have utilized crosslinked hard beads; however, here we show that channel current measurement can be achieved more simply and efficiently using non-crosslinked soft beads. In this study, we detailed the process of immobilizing channel proteins on the surface of non-crosslinked beads through chemical modification, allowing us to measure their channel activity. This method enables current measurements without the need for stringent bead size selection or high negative pressure.

**Graphical abstract:**

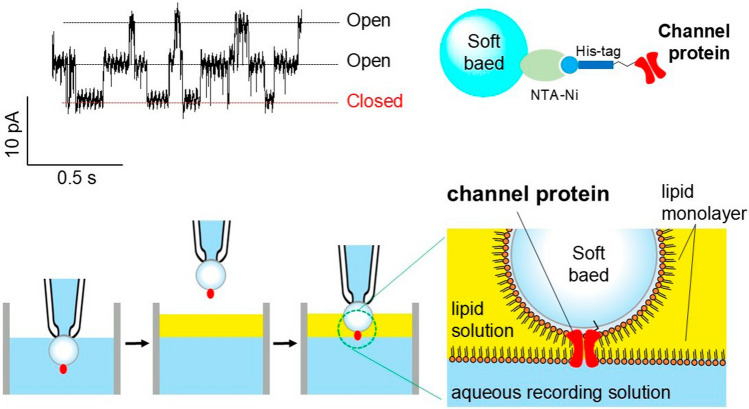

**Supplementary Information:**

The online version contains supplementary material available at 10.1007/s44211-025-00792-y.

## Introduction

Ion channel proteins are membrane proteins that regulate various biological functions by controlling the flow of ions through the cell membrane in response to stimuli. Although biological membranes are composed of a lipid bilayer and are electrically insulating, ion channels create pathways for ions by opening “holes” in the biological membrane. Ion channels have “ion selective filters” that identify ions and “sensors” that respond to various stimuli. The sensors are linked to a door called a “gate” that controls the opening and closing of the channel [1, 2].

Channel proteins are important targets for drug discovery. This is because they play critical roles in various cellular activities, and their dysregulation often leads to severe diseases. Diseases caused by abnormalities in channel proteins are collectively referred to as channelopathies, and their variety and number are vast [3]. For example, in the nervous system, epilepsy is caused by abnormalities in sodium channels, in the cardiovascular system, atrial fibrillation is linked to potassium-channel dysfunction, and in the respiratory system, cystic fibrosis is associated with chloride channel abnormalities, affecting various organs. Consequently, the development of new drugs targeting channel proteins has been actively pursued across a wide range of disease areas, including the nervous and cardiovascular systems.

Progress in drug discovery targeting channel proteins has been slow due to the lack of suitable screening devices for identifying seed compounds. For example, in drug discovery targeting water-soluble enzymes, enzyme activity can be easily measured in aqueous solutions. In contrast, drug discovery targeting channel proteins requires special measurement systems for handling membrane proteins, which is time-consuming and slows the pace of discovery. Therefore, we developed a high-efficiency channel activity measurement device to support drug discovery.

The “artificial lipid planar membrane method,” a method for measuring the ion transport activity of channel proteins with high sensitivity and resolution, reconstitutes channel proteins in an artificially fabricated lipid bilayer membrane, inserts electrodes into two chambers separated by the lipid bilayer membrane, and measures the ion flow as a current [4, 5]. However, this method takes time to measure the channel current due to the low efficiency of the process of forming the lipid bilayer membrane and the subsequent process of incorporating the channel protein into the lipid bilayer.

Efforts have been made to simplify the formation of lipid bilayer membranes. For example, the droplet contact method (DCM) [6–8] and contact bilayer method (CBB) [9, 10] measure single-channel currents by quickly forming lipid bilayer membranes at the droplet–droplet interface in a lipid solution. A multi-channel simultaneous measurement device using DCM has been developed to improve the measurement efficiency. We have also proposed a method for rapidly forming artificial membranes by bringing lipid solutions into contact with gel plates [11]. Furthermore, we have reported that inserting agarose beads into a lipid-solution layer from a water solution causes a bilayer to form spontaneously on the agarose beads [12]. This bilayer formation technology has made it possible to create artificial bilayers for channel recording in a much shorter time than with conventional techniques.

On the other hand, various developments are being made to improve the efficiency of incorporating channel proteins into lipid bilayer membranes. Although some approaches mechanically introduce channel proteins using hydrogel probes [13], we succeeded in increasing the incorporation efficiency of channel proteins by pressing the lipid bilayer membranes against gel beads with immobilized channels [14]. Furthermore, we also succeeded in accelerating the formation of artificial lipid bilayer membranes and the incorporation of channel proteins into artificial lipid bilayers by immobilizing channel proteins on an AFM [15] probe or PEG-modified gold electrodes [16] and bringing the lipid monolayer formed on the surface of these materials into contact with the lipid monolayer formed at the interface between the lipid solution and the recording solution.

In this paper, we report further improvements to our previously developed methods, which have been made to achieve greater measurement efficiency and increased versatility, so that they can be applied to a wide range of channels.

## Experimental

### Materials

Asolectin, Sepharose 6B, and Glycidol were purchased from Sigma-Aldrich (St. Louis, MO, USA). Epoxy-activated Sepharose 6B was obtained from Cytiva (Uppsala, Sweden). 2-Picoline borane was purchased from Junsei (Tokyo, Japan) and AB-NTA free acid was obtained from Dojindo (Kumamoto, Japan). Streptavidin, Alexa Fluor 546 conjugate was purchased from Invitrogen (Eugene, OR, USA). *n*-Decane and Nickel (II) chloride were purchased from Wako (Osaka, Japan).

### Processing of a glass pipette

A glass pipette (GC150T-10; Harvard Apparatus, Hollison, MA, USA) was pulled using a pipette puller (P-97; Sutter Instruments, Novato, CA, USA) to form a thin pipette (< 1 µm). Then, the tip was cut to increase the aperture diameter and polished using a microforge (MF-900; Narishige, Tokyo, Japan) to smooth the surface of the tip.

### Chemical modification of agarose gel beads

NTA or avidin-agarose beads_(12)_: AB-NTA (2.5 mg/mL) or Streptavidin (50 µg/mL) in 0.1 M borate buffer (pH 10.5) (2 mL) was added to epoxy-activated Sepharose 6B beads (500 µL, wet) and shaken slowly at room temperature overnight. Afterward, 0.5 M ethanolamine was added to the reaction mixture to block the free epoxy groups. The products were then washed thoroughly with Milli-Q water, resulting in either NTA or avidin beads [17]. The NTA beads (500 µL, wet) were immersed in 50 mM NiCl₂ solution (2 mL) to coordinate Ni^2^⁺.

NTA or avidin-agarose beads_(2)_: NaBH₄ (2 mg/mL) in 1 M NaOH (10 mL) was added to Sepharose 6B (2 mL, wet) in Milli-Q water and glycidol was added dropwise to the suspension of beads at < 25 °C. The mixture was shaken slowly at room temperature overnight. After washing thoroughly with Milli-Q water and 1 M NaCl, 0.1 M NaIO₄ (2 mL) was added to the bead suspension and shaken slowly at room temperature for 1 h. The glyoxyl-agarose beads were thus obtained.

The glyoxyl-agarose beads (2 mL, wet) were added to AB-NTA (2.5 mg/mL) or streptavidin (50 µg/mL) in MeOH-AcOH (10:1) (8 mL). Pic-BH₃ (10 mg/mL) was then added to the suspension, which was shaken slowly at room temperature for 24 h. After the products were washed thoroughly with Milli-Q water, the NTA or avidin beads were obtained [18–20]. The NTA beads (2 mL, wet) were immersed in 50 mM NiCl₂ solution (8 mL) to coordinate Ni^2^⁺.

### Expression and purification of KcsA channels

The pQE-30 plasmids containing the KcsA channel gene having a histidine tag (His-tag) with/without an Avi-tag were transformed into *Escherichia coli* XL1-Blue and the KcsA channel protein was overexpressed in 0.5 mM isopropyl-β-D(–)-thiogalactopyranoside (IPTG). The overexpressed protein was extracted from the membrane fractions using 10 mM *n*-decyl-β–D-maltoside (DM). The extracted channel protein solution was then mixed with Co^2+^ affinity gel beads (TALON Metal Affinity Resins; Takara Bio, Japan) and incubated for 30 min at 4 ℃ to bind the His- tag to the resin. Non-specifically bound proteins were removed using a washing buffer (5 mM DM, 100 mM KCl, 20 mM Tris–HCl (pH 7.5), and 20 mM imidazole). Finally, the channel proteins were eluted using a buffer (5 mM DM, 100 mM KCl, 20 mM Tris–HCl (pH 7.5), and 400 mM imidazole).

### Fluorescence microscopy

Fluorescence imaging was performed using an Olympus CKX41 inverted fluorescence microscope equipped with a U-RFLT50 mercury lamp. A filter set consisting of a BP460-490C excitation filter, DM500 dichroic mirror, and BA520IF emission filter was used to selectively excite and detect EGFP fluorescence signals.

### Electrophysiology

Ion currents across the bilayer membranes were measured with a patch-clamp amplifier (CEZ2400, Nihon-Kohden, Japan). Currents were digitized with a Digidata 1550 B (Molecular Devices, USA) and sampled at 10 kHz using the pCLAMP 10 software (Molecular Devices, USA). Membrane potential was defined as the voltage on the upper side of the membrane with respect to the bath side held at virtual ground. Channel currents were analyzed using the Clampfit 10.7 software (Molecular Devices, USA).

## Results and discussion

### Formation of bilayers on an agarose gel bead

**Fig. 1:**
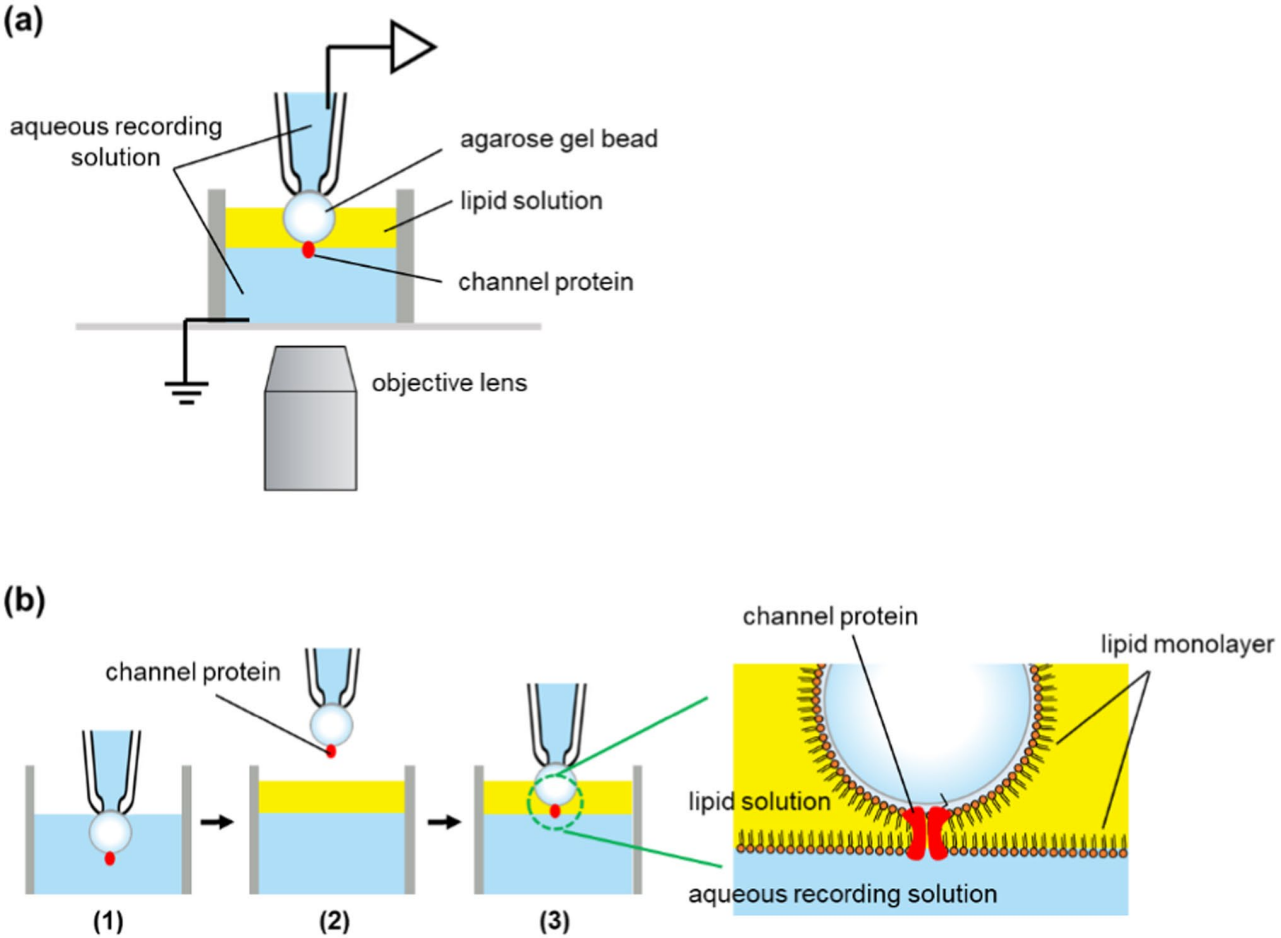
**a** The experimental apparatus. An agarose gel bead was fixed at the glass pipette tip by suction and the recording solution in the pipette was connected to the patch-clamp amplifier through an Ag–AgCl electrode. A cut glass tube (8 mm in diameter) was used as the bath chamber. **b** Bilayer formation process. (1) A gel bead was fixed at the pipette's tip by suction. (2) The bead was lifted into the air and the lipid solution was layered onto the recording solution. (3) The bead was plunged into the recording solution through the lipid solution

Artificial bilayer membranes were fabricated at the interface between an agarose gel bead and an aqueous recording solution. Figure 1a shows a schematic diagram of how to form a bilayer. A glass tube with an inner diameter of 8 mm was used as a bath chamber. In the aqueous recording solution in the bath chamber, agarose beads with channel proteins were fitted to the tip of a glass pipette by suction. The gel bead was lifted up into the air, and the lipid solution (30 mg/mL asolectin in *n*-decane or hexadecane) was layered onto the recording solution. The gel bead was then pushed down through the lipid solution to the interface between the aqueous recording solution and the lipid solution. At this point, the lipid monolayer formed on the surface of the gel bead and the lipid monolayer at the interface of the recording solution came into contact to form an artificial lipid bilayer membrane. The channel protein immobilized on the surface of the gel bead was incorporated into the artificial lipid bilayer after its formation (Fig. 1b).

### Suction fixation of an agarose gel bead at the tip opening of a glass pipette

**Fig. 2:**
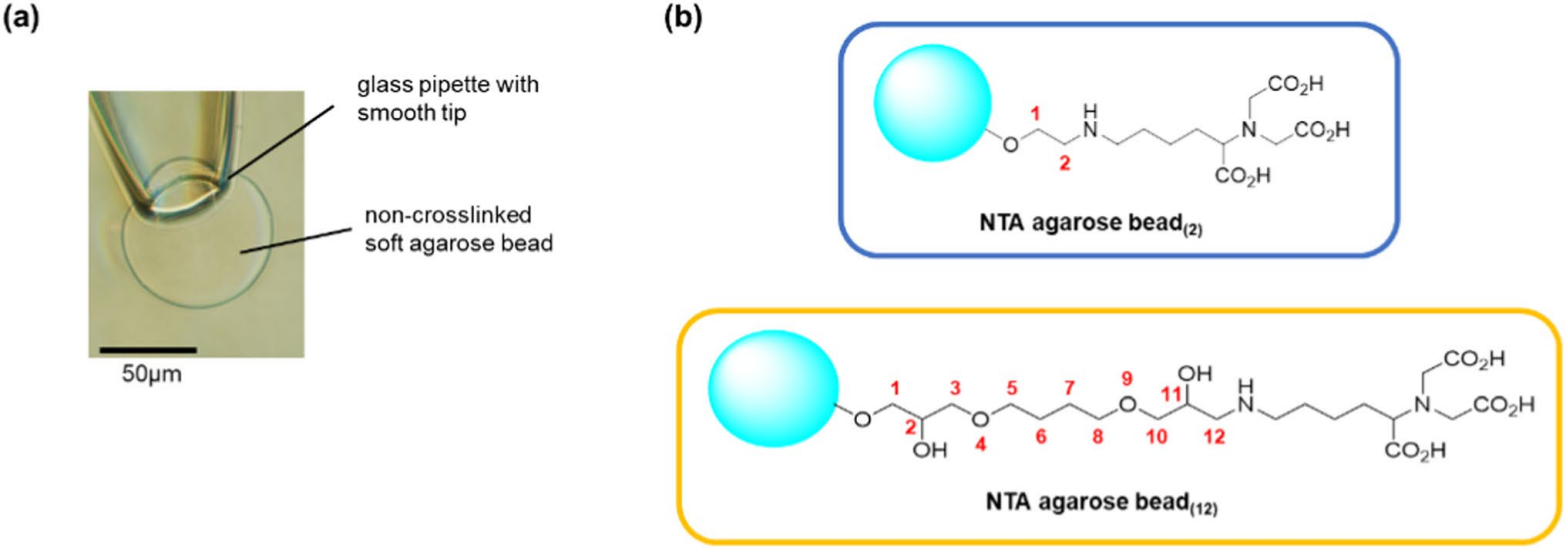
**a** The soft agarose bead was fitted into the smooth tip of a thin glass pipette. Suction deforms the bead, which maintains tight contact with the glass surface. **b** Two types of Ni–NTA agarose beads with different lengths of the spacer. The beads were made of non-crosslinked agarose beads by chemical modification

The gel bead with channels was suction-fixed to the tip of a glass pipette. It was important to tightly fix the bead to the pipette to prevent a gap between the gel bead and the pipette surface (Fig. 2a). When fixing the bead to the glass pipette opening, the bead must be in full contact with the pipette opening. If there is even a small gap, the lipid solution would flow into the pipette and interfere with the formation of a stable artificial bilayer membrane. We made “Soft” non-crosslinked agarose gel beads to hold tagged proteins, because commercially available crosslinked affinity resin beads were hard and inflexible to fit in the glass pipette tip. The non-crosslinked gel beads with immobilized channels could fit onto the tip of a glass pipette, and we successfully measured the channel current using the method shown in Fig. 1b. The tip of the glass pipette had an inner diameter of around 30 µm and gel beads with a diameter of around 80–100 µm were fixed in place. As shown in Fig. 2a, the soft bead deformed slightly upon aspiration and tightly adhered to the pipette tip, thereby preventing the lipid solution from flowing between the bead and the glass pipette.

In our previous study, we performed similar measurements using commercially available crosslinked agarose beads (TALON; Takara Bio, Japan), which were used for protein affinity purification. The advantage of using commercially available crosslinked beads is that they are easy to prepare. However, a significant drawback is that the bead size must be carefully chosen to ensure a tight seal between the pipette surface and the bead. When using crosslinked beads, it was necessary to select the bead size meticulously and apply strong suction (− 0.08 MPa) during use. Non-crosslinked soft agarose beads were used in this study. This choice allowed us to fix the gel beads to the pipette without the need for careful selection of bead size. Even with weak suction (− 0.02 MPa), the soft agarose beads were firmly sealed to the tip of the glass pipette, effectively preventing any lipid inflow through the gap between the bead and the glass pipette.

### Immobilization of channel proteins on an agarose gel bead

**Fig. 3:**
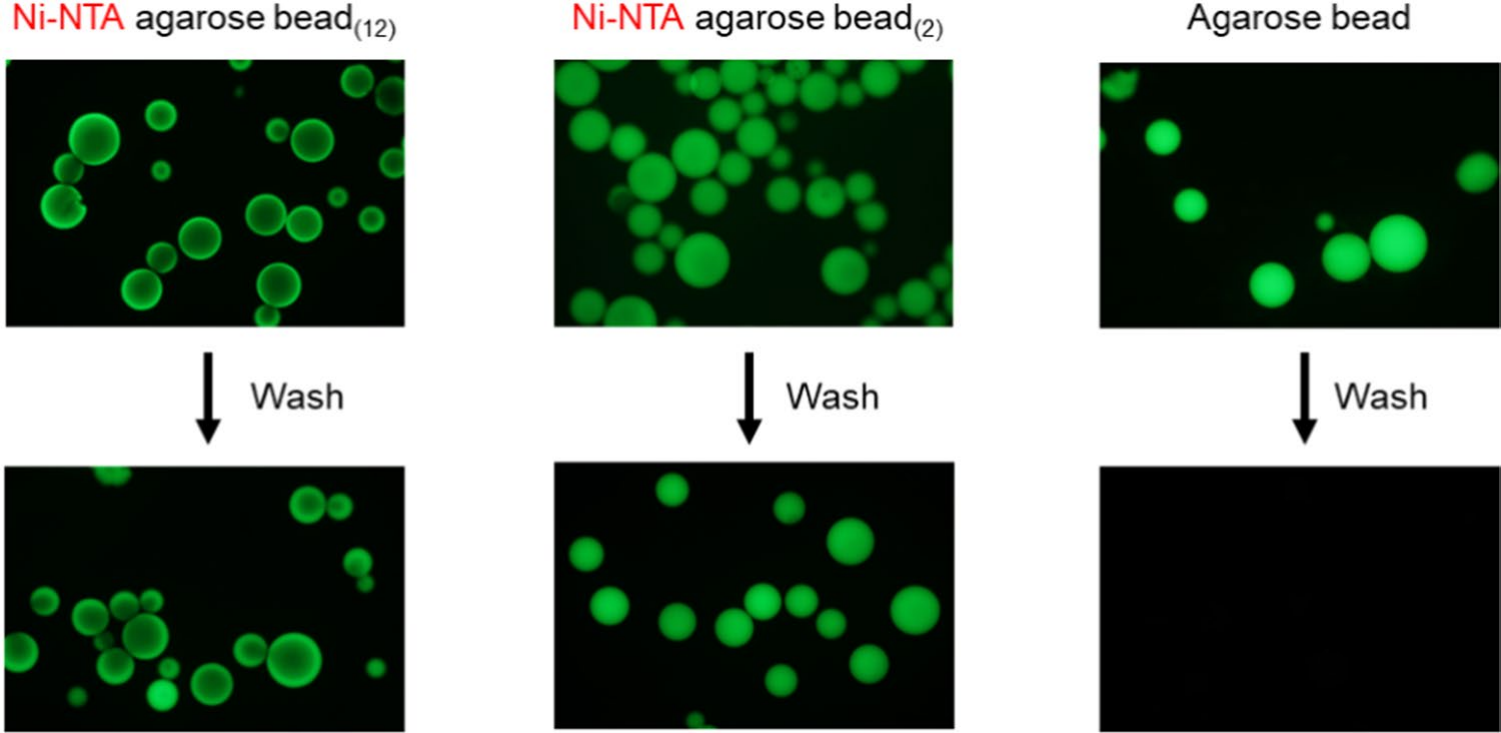
Ni–NTA agarose beads by chemical modifications hold His-tagged EGFP. The chemically modified beads were mixed with the solution containing EGFP proteins. After several washes with a solution containing 150 mM NaCl, 20 mM Tris–HCl (pH 7.5), and 20 mM imidazole, EGFP proteins were retained on the surface of only Ni–NTA agarose beads. The left image shows fluorescence microscopy of Ni–NTA-agarose beads with long spacers, the middle image features beads with short spacers, and the right image displays unmodified beads. The excitation light wavelength was 460–490 nm

Two types of agarose beads with different spacer lengths were prepared from non-crosslinked agarose beads via chemical modification (Fig. 2b). Modifying the gel bead surface with NTA and coordinating Ni^2+^ enables the immobilization of channel proteins with His-tags. To confirm that the surfaces of the beads were modified, EGFP with a His-tag was bound to the modified beads, and it was found that EGFP bound to the entire surface of the beads in both cases (Fig. 3). This indicates that the entire bead surface was correctly modified. We also prepared beads with streptavidin-modified surfaces. Biotin-tagged proteins can be immobilized on the surface of these beads.

In the fluorescence image shown in Fig. 3, the position of the fluorescence in the gel bead differs depending on the length of the spacer. We speculated that the difference in spacer length, corresponding to approximately ten carbon atoms, caused a variation in the distance between the bead surface and the protein (i.e., the thickness of the water layer formed on the bead surface). As a result, the density of the protein immobilized on the beads may have differed, leading to the observed differences in the fluorescent positions.

### Current recordings of the KcsA channel on an agarose gel bead

In this study, we conducted experiments using the KcsA E71A mutant as the measurement material. The KcsA channel, a small potassium channel from *Streptomyces lividans*, consists of approximately 160 amino acids. It forms a tetramer, creating a potassium ion-conducting pore that opens in acidic conditions (pH 4.0–5.5) and remains closed at neutral or higher pH. The KcsA E71A mutant opens more easily than the wild type and exhibits reduced C-type inactivation [21, 22].

To immobilize the KcsA E71A protein, which features a His-tag at the N-terminus, we combined the KcsA solution with approximately 10 µL of wet agarose beads, resulting in a final KcsA amount of 5 µg. After the initial mixing, the agarose beads were washed to eliminate any unbound KcsA. We then transferred the beads from the lipid solution layer, which contained 30 mg/mL asolectin in *n*-decane, to the interface with a measurement solution composed of 200 mM KCl and 10 mM succinic acid (pH 4.0), according to the procedure outlined in Fig. 1b.

**Fig. 4:**
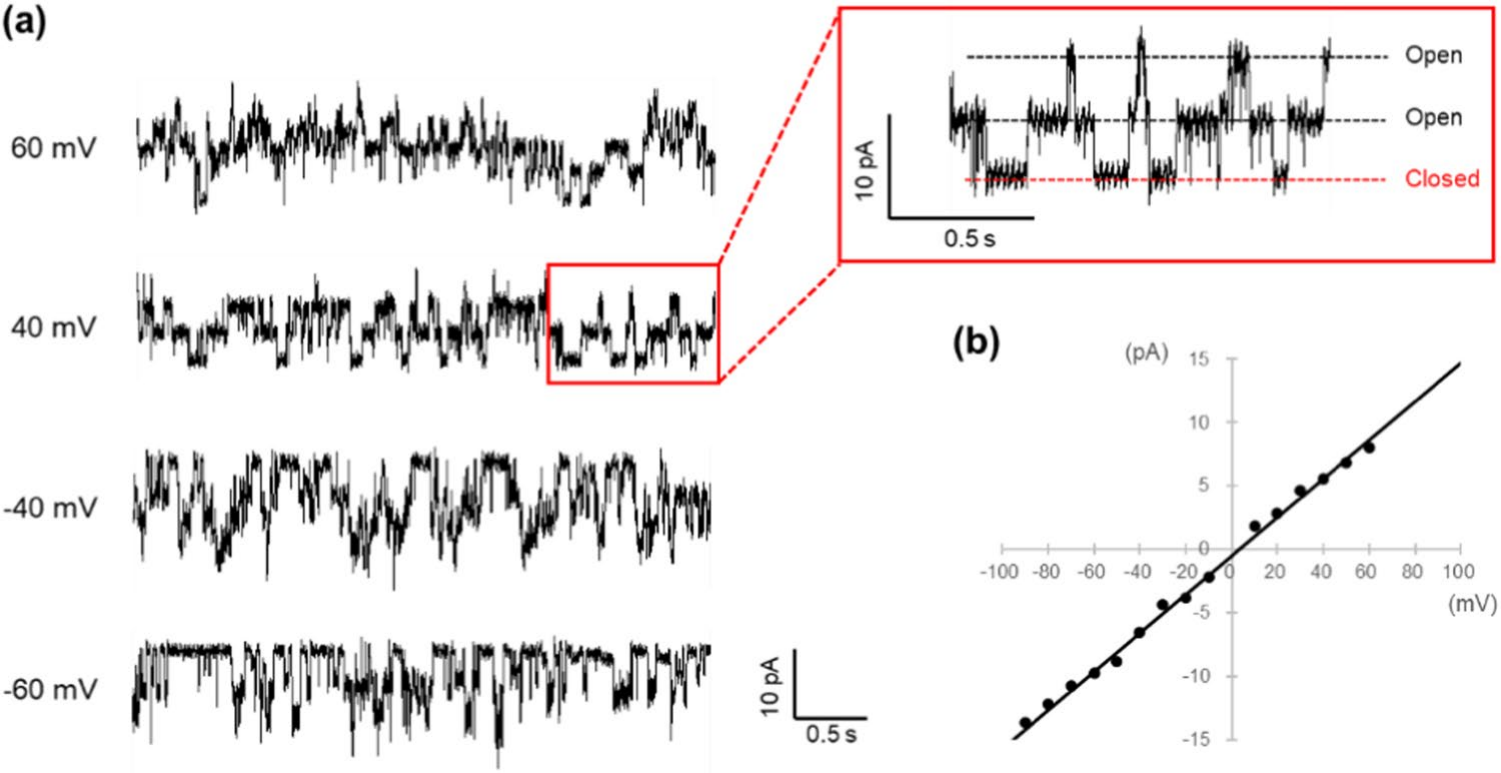
**a** Channel current recording for KcsA E71A, which has an N-terminal His-tag attached to a Ni–NTA agarose bead. The recording solution consisted of 200 mM KCl and 10 mM succinic acid at pH 4.0. Each trace shows the current fluctuation recorded at the indicated membrane potential. Each trace represents the current fluctuations recorded at the indicated membrane potential. The current trace highlighted in the red box in the upper right is an enlarged view of the red box in the left figure. At least two channel proteins are incorporated into the membrane. **b** I–V relation of KcsA E71A N-His. The single-channel conductance was determined to be 152 pS from the slope of the line

Within a few minutes of contact with the lipid–aqueous solution interface, we detected channel currents from KcsA (Fig. 4). This observation indicated that a lipid bilayer membrane was established at the interface between the agarose surface and the measurement solution layer, facilitating the incorporation of KcsA immobilized on the agarose beads into the lipid bilayer membrane.

Figure 4a presents the current traces of KcsA E71A with a His-tag attached to the N-terminus, which was anchored to the agarose beads. Each trace reflects current fluctuations at an indicated potential. In this experiment, it was estimated that two or three channels were incorporated into the membrane. The I–V relationship for a single channel derived from these traces is illustrated in Fig. 4b, and from the slope of this graph, the single-channel conductance is calculated to be 154 ± 8 pS (*n* = 7). This value aligns well with the conductance obtained using the conventional artificial bilayer membrane method, suggesting that this new approach can effectively measure channel currents comparable to standard methods.

Furthermore, when a channel protein with a C-terminal His-tag was immobilized, the channel current was measured similarly, yielding a single-channel conductance of *γ* = 150 ± 7 pS (*n* = 7). His-tagged KcsA was successfully incorporated into the lipid bilayer regardless of whether it was attached to the N or C-terminus.

We also investigated the effect of spacer length on the surface of the gel beads, but regardless of which beads were used, KcsA was incorporated into the lipid bilayer and KcsA channel currents were observed. When a relatively small protein such as KcsA was immobilized on the gel beads, no difference in the length of the spacer functional group on the surface of the beads was observed (Fig. S1).

Although billions of spacer functional groups are believed to exist on the surface of agarose beads [18], the results indicated that only a few KcsA channels were observed. This suggests that many of the KcsA channels anchored to the gel bead surface were not effectively reconstituted into the artificial membrane. It is possible that they failed to reach the lipid bilayers that were formed on the bead surface or that the orientation of the channel molecules was not conducive to reconstitution.

**Fig. 5:**
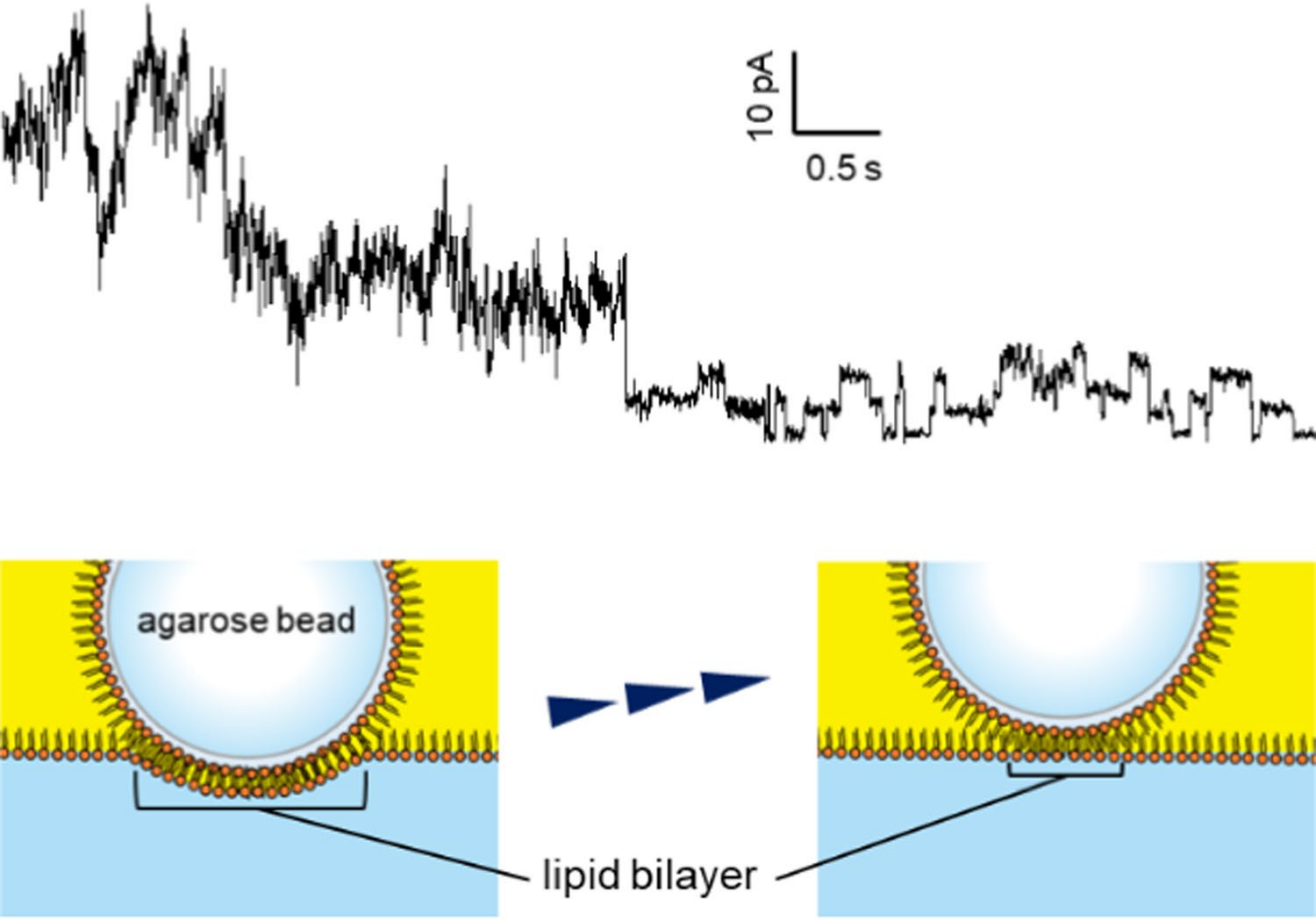
The current of KcsA E71A on Ni–NTA agarose bead at 40 mV. The upward movement of the agarose bead resulted in a reduction in the number of observed channels. By moving the bead, the channel proteins were covered by the thick lipid membrane, and the flowing current decreased

Moreover, when a substantial number of channels were incorporated into the artificial bilayer membrane, moving the agarose beads upwards led to a reduction in the number of channels observed (Fig. 5). The artificial lipid bilayer developed on the gel beads was surrounded by a thick annulus, similar to planar bilayers produced using the conventional methods. In our approach, the area of the lipid bilayer membrane can be modified based on the depth of the beads submerged in the lipid–solution interface. A larger bilayer could be achieved by pushing the beads further down. Reduction in the number of observed channels was likely due to the upward movement of the beads, which decreased the membrane area and caused a thick annular structure to cover the channels that had been reconstituted near the edges of the bilayer membrane, thereby preventing current from flowing through them.

The vertical (z-axis) movement of the bead was performed using a manual micromanipulator (One-axis Oil Hydraulic Micromanipulator: MMO-220C, Narishige, Japan), but precise and minute control of displacement was technically difficult with this setup. Although we did not quantitatively investigate the correlation between the bead’s displacement and the number of incorporated channels, it appeared that pushing the bead down through the lipid solution expanded the interface downward, forming a large lipid bilayer at the contact area between the bead and the interface and allowing multiple channels to be inserted simultaneously. It was possible to gradually lift the bead and transition to single-channel recordings; however, we were unable to precisely control the number of incorporated channels based solely on z-directional displacement. In some cases, when the manipulator was incrementally moved by approximately 5 µm at a time, we observed a sudden decrease in the number of channels at a certain point, as shown in Fig. 5. This behavior likely reflects the fact that the measured currents originated from only a small number of proteins and that the insertion sites of the channels on the bead surface were not uniformly distributed. Therefore, the number of channels incorporated could not be reliably predicted by the bead’s position alone.

**Fig. 6:**
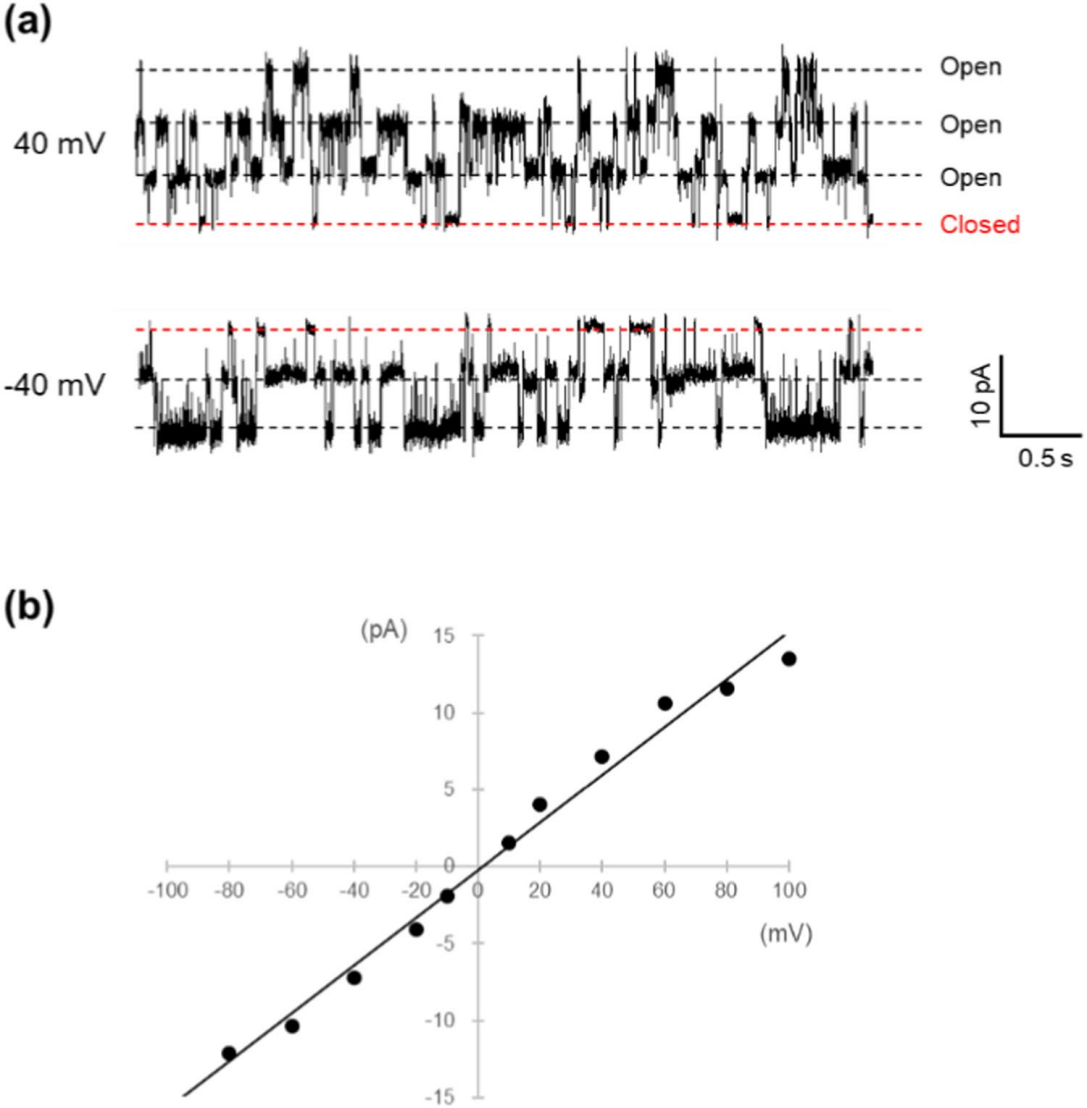
**a** Single channel recording of KcsA E71A biotin (Avi-tag) on avidin-agarose bead. The recording solution contained 200 mM KCl and 10 mM succinic acid at pH 4.0. **b** I-V relation of KcsA E71A biotin (Avi-tag). The single-channel conductance was determined to be 155 pS from the slope of the line

The current traces in Fig. 6 were acquired by reconstituting channels immobilized on agarose gel beads through avidin–biotin interactions into an artificial bilayer membrane. Specifically, KcsA channels with a biotinylated Avi-tag at the C-terminus were bound to the surface of agarose beads, which had been modified with streptavidin, as detailed in the Methods section. The channels were incorporated into the artificial membrane using the same method employed for the His-tagged proteins. Fluctuations in the current through the membrane were observed within minutes of bringing the beads into contact with the lipid-solution interface. The traces shown in Fig. 6 suggest that this channel was successfully reconstituted into the membrane. Figure 6b illustrates the I–V relationship for a single channel derived from the current recorded in Fig. 6a. From this graph, the conductance of the single channel was determined to be 150 ± 4 pS (*n* = 5).

In the conventional methods, KcsA is typically reconstituted into liposomes and subsequently incorporated into membranes via membrane fusion. This membrane fusion process, being stochastic and difficult to control, can exhibit prolonged durations, occasionally extending to tens of minutes or more [23]. The method introduced in this study allowed KcsA channels to be quickly incorporated into a bilayer membrane without the need for reconstitution into vesicles. This was achieved by immobilizing KcsA on agarose beads using either a His-tag or a biotin (avi-tag). It is presumed that the KcsA proteins immobilized on the beads were maintained in a solubilized state within the aqueous layer formed on the bead surface. During membrane formation, we believe that the detergent molecules are replaced by lipid molecules that constitute the artificial membrane, allowing KcsA to be incorporated into the membrane simultaneously. Once inserted, the KcsA channels are considered to remain stably embedded in the membrane, provided that there are no significant changes in the membrane area. Furthermore, since the channel was anchored to the gel beads, its orientation within the membrane was accurately controlled. In traditional approaches that involved freestanding planar bilayer membranes formed in aqueous solutions, the purified channels were typically reconstituted into vesicles. The vesicles were subsequently fused with a planar bilayer membrane to integrate the channels. However, this process does not guarantee a consistent orientation of the channel, as the alignment can vary once the channel is reconstituted into the vesicle membrane. In contrast, the method detailed in this study ensures that the tagged portion of the protein—specifically, the intracellular side of KcsA—remains oriented toward the bead side. This allows for reliable control over the protein's orientation in the artificial membrane.

To evaluate the orientation of the KcsA channels in the bilayer, we analyzed the effect of tetraethylammonium (TEA) on the activity of wild-type KcsA channels. In this study, the N-terminus of the KcsA WT channels was immobilized on the gel bead. Therefore, TEA was applied to the extracellular side of the channel, which was opposite to the side attached to the gel bead. The observed effect of TEA, consistent with the previous reports [24] that it only had an effect from outside the cell, supports the conclusion that the channels were incorporated into the membrane with a consistent orientation (Fig. S2).

The process of fixing the bead to the glass pipette was observed under a microscope, ensuring that bead attachment was 100% successful. Since the bead rarely detached during the measurements, the success rate of fixation was approximately 100%. As previously reported [12], bringing the bead into contact with the interface between the lipid solution and the measurement solution readily results in the formation of a lipid bilayer. While this bilayer formation was highly reproducible, the success rate of obtaining measurable channel currents was variable. Although this study did not determine the conformation of the channel protein immobilized on the beads, it is likely that the inability to obtain channel currents was not a result of channel denaturation during measurement. Instead, it seems more probable that issues arose during sample preparation. Indeed, even when experiments were conducted using the same lot of samples, the success rate varied between 50 and 90%, depending on the measurement date.

## Conclusions

We have developed a method for measuring channel currents using artificial lipid bilayers formed on agarose beads. By enhancing our previously reported method, we have significantly improved the measurement efficiency using softer beads. This new approach eliminates the need for stringent bead size selection or high negative pressure. Our method shows that an artificial lipid bilayer is rapidly formed by moving agarose beads and that channel proteins on the beads are incorporated into the lipid bilayer immediately after membrane formation. Specifically, this method addresses two major drawbacks of conventional artificial membrane techniques: the prolonged time required for membrane formation and channel incorporation. Additionally, the gel beads used in our method have a diameter of just 80–100 µm, resulting in a smaller measurement device compared to the conventional methods. These beads are also easily replaceable, enabling simple repetition of measurements, unlike traditional methods that necessitate complicated procedures such as replacing or washing the chamber after each measurement. Using this method, we have successfully recorded ion currents from several types of channel proteins. While these results demonstrate the potential versatility of the technique, further testing with a broader range of ion channels is necessary to fully evaluate its general applicability. We are currently developing a system capable of simultaneous multi-channel measurements to facilitate high-throughput recordings. By integrating this technique with microfluidic technology, we anticipate the potential to automate devices capable of simultaneously measuring multiple channel currents in parallel.

## Supplementary Information

Below is the link to the electronic supplementary material.

Supplementary file1 (DOCX 96 KB)

## Data Availability

Data for this article are available from the corresponding author upon reasonable request.

## References

[1] B. Hille, *Ion channels of excitable membranes*, 3rd edn. (Sinauer Associates Inc, Sunderland, MA, USA, 2001)

[2] J.N.C. Kew, C.H. Davies, *Ion channels: from structure to function*, 2nd edn. (Oxford University Press, Oxford, UK, 2010)

[3] C.A. Hubner, T.J. Jentsch, Ion channel diseases. Hum. Mol. Genet. **11**(20), 2435–2445 (2002). 10.1093/hmg/11.20.2435 12351579 10.1093/hmg/11.20.2435

[4] C. Miller, *Ion Channel Reconstitution* (Plenum Press, New York, 1986)

[5] T. Osaki, H. Suzuki, B.L. Pioufle, S. Takeuchi, Multichannel simultaneous measurements of single-molecule translocation in alpha-hemolysin nanopore array. Anal. Chem. **81**(24), 9866–9870 (2009). 10.1021/ac901732z 20000639 10.1021/ac901732z

[6] K. Funakoshi, H. Suzuki, S. Takeuchi, Lipid bilayer formation by contacting monolayers in a microfluidic device for membrane protein analysis. Anal. Chem. **78**(24), 8169–8174 (2006). 10.1021/ac0613479 17165804 10.1021/ac0613479

[7] H. Bayley, B. Cronin, A. Heron, M.A. Holden, W.L. Hwang, R. Syeda, J. Thompson, M. Wallace, Droplet interface bilayers. Mol. Biosyst. **4**(12), 1191–1208 (2008). 10.1039/B808893D 19396383 10.1039/b808893d PMC2763081

[8] R. Kawano, Y. Tsuji, K. Sato, T. Osaki, K. Kamiya, M. Hirano, T. Ide, N. Miki, S. Takeuchi, Automated parallel recordings of topologically identified single ion channels. Sci. Rep. **3**, 1995 (2013). 10.1038/srep01995 23771282 10.1038/srep01995 PMC3683667

[9] W.L. Hwang, M. Chen, B. Cronin, M.A. Holden, H. Bayley, Asymmetric droplet interface bilayers. J. Am. Chem. Soc. **130**(18), 5878–5879 (2008). 10.1021/ja802089s 18407631 10.1021/ja802089s

[10] M. Iwamoto, S. Oiki, Contact bubble bilayers with flush drainage. Sci. Rep. **5**, 9110 (2015). 10.1038/srep09110 25772819 10.1038/srep09110 PMC4360637

[11] T. Ide, T. Kobayashi, M. Hirano, Lipid bilayers at the gel interface for single ion channel recordings. Anal. Chem. **80**(20), 7792–7795 (2008). 10.1021/ac801224a 18800849 10.1021/ac801224a

[12] M. Hirano, D. Yamamoto, M. Asakura, T. Hayakawa, S. Mise, A. Matsumoto, T. Ide, A lipid bilayer formed on a hydrogel bead for single ion channel recordings. Micromachines **11**(12), 1070 (2020). 10.3390/mi11121070 33271761 10.3390/mi11121070 PMC7759777

[13] M.A. Holden, H. Bayley, Direct introduction of single protein channels and pores into lipid bilayers. J. Am. Chem. Soc. **127**(18), 6502–6503 (2005). 10.1021/ja042470p 15869249 10.1021/ja042470p

[14] M. Hirano, Y. Takeuchi, T. Aoki, T. Yanagida, T. Ide, Current recordings of ion channel proteins immobilized on resin beads. Anal. Chem. **81**(8), 3151–3154 (2009). 10.1021/ac900286z 19296686 10.1021/ac900286z

[15] M. Kitta, T. Ide, M. Hirano, H. Tanaka, T. Yanagida, T. Kawai, Direct manipulation of a single potassium channel gate with an atomic force microscope probe. Small **7**(16), 2379–2383 (2011). 10.1002/smll.201002337 21656673 10.1002/smll.201002337

[16] D. Okuno, M. Hirano, H. Yokota, J. Ichinose, T. Kira, T. Hijiya, C. Uozumi, M. Yamakami, T. Ide, A gold nano-electrode for single ion channel recordings. Nanoscale **10**(8), 4036–4040 (2018). 10.1039/c7nr08098k 29431813 10.1039/c7nr08098k

[17] L. Sundberg, J. Porath, Preparation of adsorbents for biospecific affinity chromatography: I. Attachment of group-containing ligands to insoluble polymers by means of bifunctional oxiranes. J. Chromatogr **90**(1), 87–98 (1974). 10.1016/S0021-9673(01)94777-6 4824665 10.1016/s0021-9673(01)94777-6

[18] J.M. Guisan, Aldehyde-agarose gels as activated supports for immobilization-stabilization of enzymes. Enzyme Microb. Technol. **10**(6), 375–382 (1988). 10.1016/0141-0229(88)90018-X

[19] F. López-Gallego, G. Fernandez-Lorente, J. Rocha-Martin, J.M. Bolivar, C. Mateo, J.M. Guisan, Stabilization of enzymes by multipoint covalent immobilization on supports activated with glyoxyl groups. Methods Mol. Biol. **1051**, 59–71 (2013). 10.1007/978-1-62703-550-7_5 23934798 10.1007/978-1-62703-550-7_5

[20] S. Sato, T. Sakamoto, E. Miyazawa, Y. Kikugawa, One-pot reductive amination of aldehydes and ketones with a-picoline-borane in methanol, in water, and in neat conditions. Tetrahedron **60**(36), 7899–7906 (2004). 10.1016/j.tet.2004.06.045

[21] J.F. Cordero-Morales, L.G. Cullo, Y. Zhao, V. Jogini, D.M. Cortes, B. Roux, E. Perozo, Molecular determinants of gating at the potassium-channel selectivity filter. Nat. Struct. Mol. Biol. **13**, 311–318 (2006). 10.1038/nsmb1069 16532009 10.1038/nsmb1069

[22] M. Hirano, Y. Onishi, T. Yanagida, T. Ide, Role of the KcsA channel cytoplasmic domain in pH-dependent gating. Biophys. **101**(9), 2157–2162 (2011). 10.1016/j.bpj.2011.09.024 10.1016/j.bpj.2011.09.024 PMC3207171 22067153

[23] J. Zimmerberg, F.S. Cohen, A. Finkelstein, Fusion of phospholipid vesicles with planar phospholipid bilayer membranes. I. Discharge of vesicular contents across the planar membrane. J. Gen. Physiol. **75**(3), 241–250 (1980). 10.1085/jgp.75.3.241 6247417 10.1085/jgp.75.3.241 PMC2215256

[24] L. Heginbotham, M. LeMasurier, L. Kolmakova-Partensky, C. Miller, Single streptomyces lividans K+ channels: Functional asymmetries and sidedness of proton activation. J. Gen. Physiol. **114**, 551–560 (1999). 10.1085/jgp.114.4.551 10498673 10.1085/jgp.114.4.551 PMC2229473

