# Supplementary material for: Direct insertion of an ion channel immobilized on a soft agarose gel bead into a lipid bilayer: an optimized method
Source: Anal Sci. 2025 May 20;41(7):1073–82. doi: 10.1007/s44211-025-00792-y (PMC12202583; doi:10.1007/s44211-025-00792-y)
Supplement: Supplementary file 1 — Supplementary file1 (DOCX 96 KB) [file 44211_2025_792_MOESM1_ESM.docx]

**Supplementary Information**

Title:

**Direct insertion of an ion channel immobilized on a soft agarose gel bead into a lipid bilayer: an optimized method**

Journal name:

**Analytical Sciences**

Author names:

**Mami Asakura^1, 2^, Shuyan Wang^1^, Minako Hirano^1^, Toru Ide^1*^**

Affiliation:

**1 Graduate School of Interdisciplinary Science and Engineering in Health Systems, Okayama University, 3-1-1 Tsushima-naka, Kita-ku, Okayama-shi, Okayama, 700-8530, Japan**

**2 Department of Comprehensive Technical Solutions, Okayama University, 3-1-1 Tsushima-naka, Kita-ku, Okayama-shi, Okayama, 700-8530, Japan**

E-mail address of the corresponding author:

[**ide@okayama-u.ac.jp**](mailto:ide@okayama-u.ac.jp) **(Toru Ide)**


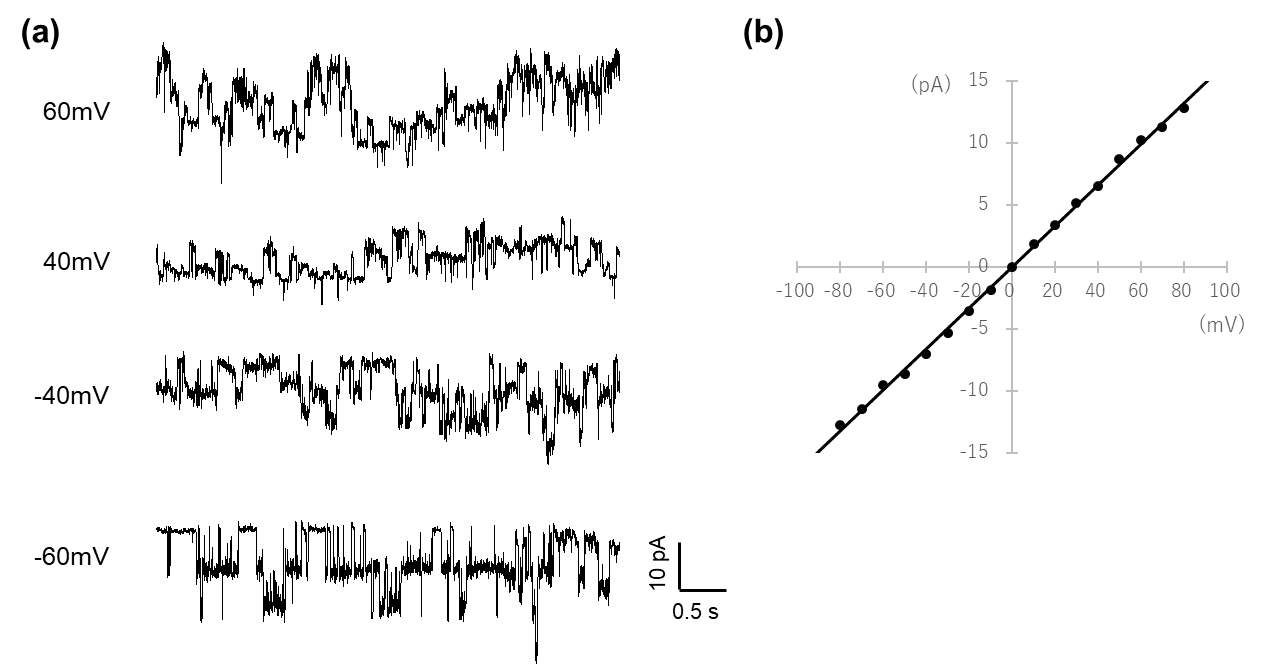


**Fig. S1**

(a) Channel current recording for KcsA E71A, which has an N-terminal His-tag attached to a Ni-NTA agarose bead_(2)_. The recording solution consisted of 200 mM KCl and 10 mM succinic acid at pH 4.0. (b) I-V relation of KcsA E71A N-His. The single channel conductance was determined to be 153 ± 14 pS (n=4).


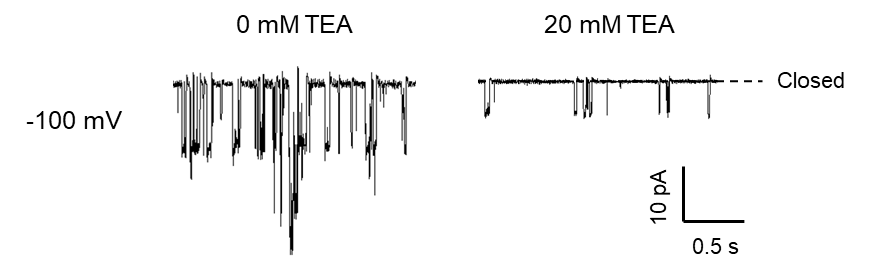


**Fig. S2**

Effects of tetraethylammonium (TEA) on the activity of wild-type KcsA, which has an N-terminal His-tag attached to a Ni-NTA agarose bead. The recording solution contained 200 mM KCl and 10 mM succinic acid at pH 4.0. TEA was applied at a final concentration of 20 mM; 0 mM TEA served as the control.
